# Analytical Entropy Approach for Measuring Blockchain Immutability and Tamper-Resilient Trust

**DOI:** 10.3390/e28060690

**Published:** 2026-06-15

**Authors:** Lanlan Li, Charles Z. Liu, Sanjeeb Shrestha

**Affiliations:** 1 School of Information Engineering, Chuzhou Polytechnic, Chuzhou 239000, China; lilanlan@chzc.edu.cn; 2 Security TEEs, AlphaNest, Sydney, NSW 2036, Australia; cliu@mit.edu.au; 3 School of IT and Engineering, Melbourne Institute of Technology, Sydney, NSW 2000, Australia; 4 The University of Notre Dame Australia, Sydney, NSW 2007, Australia

**Keywords:** blockchain, entropy, immutability, consensus mechanism, on-chain data integrity, off-chain data security, smart contracts, blockchain virtual machine, information theory, attack resilience

## Abstract

This work presents a comprehensive study of entropy-based metrics for evaluating blockchain systems, focusing on on-chain ledger immutability, off-chain data integrity, and computational dynamics within blockchain virtual machines (BVMs). We develop a unified framework that models blockchain states as probabilistic distributions, quantifying uncertainty through Shannon entropy and examining its evolution under varying adversarial fractions. Extensive simulations demonstrate that on-chain entropy exhibits near-exponential decay, reflecting the cumulative reinforcement of honest consensus, while off-chain entropy remains static, highlighting the limitations of conventional data storage. Furthermore, the BVM is analyzed in terms of computation entropy, establishing its Turing completeness and demonstrating that smart-contract state evolution mirrors the information dynamics of arbitrary Turing machines. Our results provide quantitative evidence that entropy serves as both a theoretical and operational measure of immutability, tamper evidence, and protocol resilience. The proposed entropy framework offers practical tools for monitoring ledger integrity, detecting tampering, and assessing computational complexity, bridging the gap between information-theoretic principles and distributed ledger applications. This study advances both the theoretical understanding and practical evaluation of blockchain security, providing a principled methodology for analyzing distributed systems under adversarial conditions.

## 1. Introduction

Blockchain technology constitutes a form of decentralized computational and record-keeping infrastructure wherein the interplay of computation, cryptography, and consensus protocols underpins both functionality and security [1,2]. Fundamentally, a blockchain implements a distributed ledger reinforced by cryptographic primitives that guarantee the immutability and verifiability of on-chain data, even in adversarial settings [3,4,5]. From an information-theoretic perspective, the evolution of uncertainty and entropy associated with ledger states provides a quantitative lens for analyzing trust, finality, and tamper resistance as emergent properties of consensus dynamics [6,7,8,9].

Despite widespread deployment, existing analytical frameworks are limited in capturing both the probabilistic and computational facets of blockchain security. Consensus protocols, exemplified by Nakamoto consensus, are designed to enforce globally consistent state transitions in the presence of Byzantine participants [10,11,12]. The integrity of these transitions relies on cryptographic primitives, including digital signatures, hash functions, and interlinked block structures. Together, these mechanisms contribute to a reduction in informational entropy over time, collapsing diverse potential histories toward a dominant, honest chain. However, practical architectures frequently integrate off-chain storage to manage scalability and sensitive data. In such hybrid systems, on-chain hash commitments preserve only a digest of the original data, leaving the underlying off-chain content vulnerable to undetected tampering and resulting in a differential between on-chain and off-chain entropy profiles.

The Probabilistic Polynomial-Time (PPT) adversary model provides a mathematically tractable foundation for security analysis by constraining adversaries to efficient randomized algorithms and defining negligible success probabilities for cryptographic attacks [13,14,15]. Extending PPT reasoning to distributed ledgers requires formal abstraction, and the blockchain must be modeled as a stochastic computational process with an evolving probability distribution over possible histories. In this context, entropy serves as a principled measure of uncertainty, capturing the extent to which adversarial actions can diversify the space of plausible ledger states. Analytic methods rooted in probability theory and combinatorial bounds are indispensable for quantifying adversarial success under this model. Further, entropy decay theory from stochastic processes and information theory provides formal tools for the modeling of the contraction of uncertainty over time [16,17,18,19].

From an information-theoretic perspective, the entropy rate of a stochastic process represents the average uncertainty per unit of time, and its convergence properties are well established for ergodic Markov chains and stationary processes [20,21]. In blockchain consensus, such entropy convergence captures the stabilization of ledger-state distributions: as confirmations accumulate, the probability distribution over possible forked histories contracts, and the associated entropy approaches a limiting value. This formalizes the intuitive notion that honest-majority consensus dissipates uncertainty and provides a principled metric for tamper resilience.

Although prior studies have explored entropy-related and information-theoretic perspectives in blockchain systems, existing approaches remain fragmented and are often restricted to protocol-specific analyses. In particular, previous works have investigated blockchain security through probabilistic consensus analysis, adversarial mining models, and stochastic security formulations [22,23,24,25,26,27]. Other studies have employed entropy-based and information-theoretic metrics to characterize uncertainty, randomness, transaction behavior, and distributed trust in blockchain environments [6,7,9,17]. In parallel, stochastic-process and Markov-chain formulations have been introduced to analyze consensus convergence, fork dynamics, and adversarial behavior in distributed ledger systems [12,16,21,28,29,30].

In addition, a line of research has explored information-theoretic and entropy rate-based analyses in distributed systems and cryptographic settings, focusing on uncertainty quantification, convergence behavior, and information leakage under adversarial models [16,20,21]. However, such approaches are typically developed in general stochastic or information-theoretic frameworks and are not specifically unified with blockchain consensus mechanisms. Existing entropy-related blockchain studies primarily focus on several isolated directions, including transaction entropy analysis [6,7,9], mining randomness and probabilistic mining models [31,32], probabilistic consensus security [33,34], and Markovian modeling of fork dynamics and stochastic consensus evolution [12,16,21]. However, these approaches typically treat entropy either as a descriptive statistical measure or as a protocol-level security indicator within isolated subsystems. Importantly, existing entropy-based blockchain studies do not formulate entropy as a functional over probability measure-valued ledger-state processes, nor do they jointly characterize entropy evolution, spectral contraction of consensus operators, cryptographic security, and off-chain integrity within a single, unified, stochastic framework.

In contrast to existing works that treat entropy as either a descriptive metric or a protocol-specific security indicator, the present work models blockchain evolution, itself, as a probability measure-valued stochastic process and formulates entropy as a functional over evolving ledger-state distributions induced by a consensus transition kernel. This formulation enables a unified connection between entropy evolution, stochastic consensus convergence, adversarial uncertainty, computational expressiveness, and off-chain tampering detectability within a single mathematically consistent framework.

More specifically, the existing literature generally lacks a mathematically unified framework that simultaneously:

1. Models blockchain evolution as a stochastic information process over probability distributions of ledger states;
2. Rigorously connects entropy evolution with Markovian consensus dynamics, spectral contraction behavior, and asymptotic convergence properties;
3. Integrates cryptographic immutability, probabilistic adversarial analysis, smart-contract computation, and off-chain integrity within a unified entropy-theoretic formulation.

By contrast, the present work develops a generalized, stochastic, information-theoretic framework in which blockchain consistency, entropy dissipation, computational expressiveness, and tampering detectability are analyzed within a unified, probabilistic structure induced by the consensus transition kernel.

Rather than introducing entropy merely as a descriptive statistical quantity, we formalize it as a functional over evolving probability measures associated with ledger-state stochastic processes defined on a probability space. This formulation enables explicit connections between entropy contraction, spectral properties of the induced Markov operator, adversarial uncertainty, and asymptotic ledger consistency under honest-majority assumptions.

Therefore, despite substantial progress in blockchain security, probabilistic consensus analysis, entropy-rate theory, and information-theoretic modeling, a unified, stochastic, information-theoretic framework connecting entropy evolution, Markovian consensus dynamics, computational expressiveness, cryptographic immutability, and off-chain tampering detectability remains largely undeveloped in a mathematically rigorous form.

Meanwhile, smart contracts can be formalized as algorithmic agents executed within a blockchain virtual machine (BVM) [35,36,37]. The computational entropy of a contract’s state space reflects its expressive complexity, resource constraints, and termination behavior. Drawing on Turing completeness theory [38,39,40], any Turing machine (M) can be simulated by a smart contract ($C_{M}$). This formal equivalence enables a rigorous assessment of execution soundness and computational uncertainty and situates smart-contract behavior within classical computability frameworks. Despite this foundational link, existing literature lacks systematic frameworks that integrate entropy-based analysis with formal verification of contract correctness and resource-bounded execution.

Immutability, a cornerstone of blockchain security, is often invoked heuristically. A rigorous treatment requires cryptographic assumptions such as collision resistance of hash functions and existential unforgeability under chosen message attacks (EUF-CMA) of digital signatures [41,42,43]. Within this formal setting, entropy provides a quantitative measure of uncertainty reduction: as honest consensus accrues, the entropy associated with alternative chain histories diminishes, and the probability of successful undetected tampering becomes negligible. This perspective not only aligns with traditional cryptographic proofs but also situates immutability as an observable phenomenon in the entropy trajectory of the ledger.

Off-chain data introduces additional analytical challenges. Modern systems integrate oracles, IoT feeds, and cross-chain inputs, whose integrity must be provably linked to on-chain commitments. By modeling hash commitments, trusted execution environments (TEEs), and verification mechanisms, one can define measurable bounds on tampering detection accuracy as a function of entropy differences between on-chain and off-chain state spaces [44,45,46,47,48,49]. This formalization frames off-chain integrity as a probabilistic mapping constrained by cryptographic security and execution correctness.

Consensus protocols are, themselves, probabilistic systems, and the probability of a successful chain-rewriting attack can be characterized using biased random-walk models [31,32,50,51,52,53]. These models facilitate explicit derivation of decay rates for attack success probabilities as functions of adversarial resources and finality depth, connecting entropy contraction with protocol parameters. Such quantitative bounds transform intuitive notions of security into precise, provable guarantees.

Existing threat models for blockchain often remain informal. Formal frameworks such as the Dolev–Yao model define adversarial capabilities in symbolic terms [54,55,56]. Extending these models to include TEEs and entropy-based metrics yields a unified structure for end-to-end security proofs that encompass execution integrity, cryptographic assumptions, and adversary behavior.

To address these theoretical and practical gaps, we propose an entropy-centric analytic framework that integrates on-chain consensus dynamics, formal cryptographic primitives, smart-contract computation, and off-chain tamper detection. This unified approach enables the derivation of bounds on attack probabilities, computational costs, and entropy decay, offering a principled foundation for blockchain security analysis.

Our contributions are outlined as follows. First, we develop a unified, stochastic, information-theoretic framework that models blockchain evolution as a probability measure-valued stochastic process induced by a consensus transition kernel. Within this framework, entropy is rigorously formulated as a functional over ledger-state distributions rather than as a heuristic descriptor of blockchain uncertainty. Second, we establish explicit connections between entropy evolution, Markovian consensus dynamics, and asymptotic contraction behavior, providing a probabilistic interpretation of blockchain immutability and confirmation security. Third, we formally connect smart-contract computation and blockchain virtual machines (BVMs) with entropy-based computational analysis through computability-theoretic formulations. Fourth, for off-chain data integrity, we construct cryptographically sound tampering-detection formulations combining hash commitments and trusted execution environments (TEEs), enabling probabilistic analysis of off-chain uncertainty and detectability. Finally, we integrate these components into a generalized analytical framework unifying entropy evolution, adversarial uncertainty, stochastic consensus behavior, and cryptographic consistency within a common mathematical structure.

This work synthesizes entropy-based reasoning with computability theory, probabilistic analysis, cryptography, and formal methods to provide a mathematically precise foundation for evaluating distributed ledger security and integrity, offering insights that inform both theoretical understanding and practical system design.

## 2. Preliminaries

In this section, we introduce the fundamental notations and concepts from an information-theoretic perspective. Rather than viewing a blockchain solely as a cryptographic data structure, we model it as an evolving *information system* whose security and integrity properties are characterized by uncertainty, entropy, and information consistency.

Let $B=(B_{1},B_{2},\ldots ,B_{k})$ denote a blockchain represented as an ordered sequence of blocks. Each block ($B_{i}$) is defined as

$$B_{i}=\left(D_{i},h_{i-1},\sigma_{i}\right),$$

where $D_{i}$ denotes the on-chain information content, $h_{i-1}$ is a cryptographic hash commitment to the previous block, and $\sigma_{i}$ is a digital signature ensuring authenticity.

From an information-theoretic standpoint, the blockchain induces a probability distribution over possible ledger states. $P_{k}$ denotes the distribution of ledger states of length *k*, and we measure the uncertainty of the ledger using Shannon entropy, i.e.,

$$H\left(P_{k}\right)=-\sum_{B_{k}}\mathrm{Pr}\left(B_{k}\right)\log\mathrm{Pr}\left(B_{k}\right).$$

Blockchain security mechanisms aim to asymptotically reduce this entropy, driving the ledger distribution toward concentration around a dominant history with overwhelming probability.

### 2.1. Hash Commitments and Information Consistency

The cryptographic hash function (h(·)) is modeled as a many-to-one information compression function. Its collision resistance ensures that distinct information sources remain statistically distinguishable, except with negligible probability.

Let $D_{i}^{\mathrm{off}}$ denote an off-chain data object and $h_{\text{on-chain}}=h\left(D_{i}^{\mathrm{off}}\right)$ be its on-chain commitment. We interpret the integrity of off-chain data as an *information consistency* problem between two distributions, where *P* refers to the distribution of authentic off-chain information and *Q* is the distribution induced by adversarial tampering.

Tampering detection is formalized by a verification function (Detect(·)) whose objective is to distinguish *P* from *Q*. In information-theoretic terms, detection succeeds whenever the Kullback–Leibler divergence is

$$D_{\mathrm{KL}}\left(P\;∥\;Q\right)>0.$$

Collision resistance implies that *P* and *Q* become statistically indistinguishable only with negligible probability, guaranteeing reliable information consistency across the on-chain/off-chain boundary.

### 2.2. Computation, Information Processing, and Entropy

We model computation using the classical Turing-machine abstraction. A computable function (*f*) corresponds to a transformation of input information into output information. From an entropy perspective, computation may preserve, reduce, or concentrate information, depending on its reversibility and structure.

Blockchain smart contracts executed on a Blockchain Virtual Machine (BVM) are modeled as realizations of computable functions under resource constraints. The evolution of a contract state corresponds to a stochastic information process whose entropy reflects uncertainty arising from execution paths, resource limits, and adversarial inputs.

### 2.3. Adversaries and Information Uncertainty

An adversary (A) is modeled as a probabilistic polynomial-time (PPT) information processor. The security parameter (λ) controls the adversary’s ability to reduce uncertainty via computation. Events with probability bounded by a negligible function (λ) correspond to information gains that vanish asymptotically.

Let α denote the fraction of adversarial resources. The probability of a successful ledger rewrite, i.e., $P_{\mathrm{rewrite}}\left(\alpha ,k\right)$, quantifies the residual uncertainty of the ledger state after *k* confirmations. Security is achieved when

$$\lim_{k\to \infty }H\left(P_{k}|\alpha \right)=0,$$

indicating convergence toward a unique information history.

**Definition** **1** (Probability Space of Ledger Evolution)**.** *All ledger evolutions are defined on a probability space (*$\Omega ,F,P_{\alpha }$*, where* Ω *is the set of all possible infinite ledger histories,* F *is the sigma algebra over* Ω*, and* $P_{\alpha }$ *is induced by the consensus protocol with adversarial fraction α).*

**Definition** **2** (Ledger-State Stochastic Process)**.** *The sequence of probability distributions over all admissible ledger histories after k confirmations is denoted as* ${\left\{P_{k}\right\}}_{k\geq 0}$*. The evolution of the blockchain under the consensus protocol is modeled as a discrete-time stochastic process, where each transition, i.e.,* $P_{k}\to P_{k+1}$*, captures the redistribution of probability mass among competing ledger histories due to block generation, network propagation, and adversarial actions.*

**Definition** **3** (Ledger Transition Kernel)**.** *Let* S *denote the space of all admissible ledger histories. The evolution of the ledger is governed by a stochastic transition kernel:*

$$T_{\alpha }:S\times S\to \left[0,1\right],$$

*where*

$$T_{\alpha }\left(s\to s^{\prime }\right)=\mathrm{Pr}\left(X_{k+1}^{\mathrm{ledger}}=s^{\prime }|X_{k}^{\mathrm{ledger}}=s,\alpha \right).$$

*The induced distribution satisfies*

$$P_{k+1}\left(s^{\prime }\right)=\sum_{s\in S}P_{k}\left(s\right)\;T_{\alpha }\left(s\to s^{\prime }\right).$$

**Remark** **1.** *The ledger transition kernel captures consensus dynamics, including block propagation, fork resolution, and adversarial modifications under resource fraction α.*

**Definition** **4** (Dominant Honest Chain)**.** *Let the probability distribution over ledger histories after k confirmations be*

$$P_{k}=\left\{p_{i}^{\left(k\right)}\right\}.$$

*The dominant honest chain is defined as the ledger history with maximal probability under the honest-majority assumption:*

$$p_{*}^{\left(k\right)}=\max_{i}p_{i}^{\left(k\right)}.$$

*The residual inconsistent probability mass is*

$$I_{k}=1-p_{*}^{\left(k\right)}.$$

**Definition** **5** (Entropy as a Functional over Random Variables)**.** *All entropy notions in this paper are defined in the Shannon sense. Let X be a random variable defined on a probability space (Ω,F,P). The entropy of X is defined as*

$$H\left(X\right)=-\sum_{x}P\left(X=x\right)\log P\left(X=x\right).$$

*Deterministic objects such as blocks, hashes, or files are not random variables. Entropy is therefore always defined over ensembles of possible system states.*

**Definition** **6** (System-State Random Variables)**.** *We define the following random variables:*

1.
$X_{k}^{\mathrm{ledger}}$ *is a random variable representing the ledger history at depth k, induced by the consensus protocol and adversarial behavior.*
2.
$X_{t}^{\mathrm{contract}}$ *is a random variable representing the execution state of smart contracts at time t.*
3.
$X^{\mathrm{off}}$ *is a random variable representing the off-chain data state under uncertainty or adversarial modification.*

*Each entropy term in this paper is defined as the Shannon entropy of one of these random variables.*

**Definition** **7** (Entropy-Carrying Random Variables)**.** *In this work, entropy is always defined with respect to a probability distribution over possible system states:*

1. *Ledger entropy refers to the Shannon entropy of the random variable describing possible ledger histories.*
2. *Contract-state entropy refers to the entropy of the distribution over possible execution states induced by stochastic inputs, asynchronous execution, or adversarial behavior.*
3. *Off-chain information entropy refers to the uncertainty associated with a distribution over possible off-chain data states.*
4. *Tampering-related entropy measures the uncertainty introduced into the distribution of information states after adversarial modification.*

*Deterministic objects such as a specific block, hash value, or file instance do not, themselves, possess Shannon entropy; rather, entropy is associated with the uncertainty over ensembles of such objects.*

**Remark** **2.** *Throughout this paper, entropy is interpreted strictly in the Shannon sense, i.e., as a functional defined on probability distributions over possible system states. Accordingly, statements regarding “ledger entropy”, “contract-state entropy”, or “off-chain information entropy” refer to the uncertainty associated with ensembles of possible states rather than deterministic objects themselves. This distinction is important because individual blocks, hash values, and concrete files are deterministic realizations and do not directly possess Shannon entropy.*

**Assumption** **1** (Collision-Resistant Information Binding)**.** *Let a cryptographic hash function be*

$$h:{\left\{0,1\right\}}^{*}\to {\left\{0,1\right\}}^{n}.$$

*For any probabilistic polynomial-time adversary (A), the probability of finding two distinct inputs, i.e.,*

$$x\neq x^{\prime },$$

*such that*

$$h\left(x\right)=h\left(x^{\prime }\right)$$

*is negligible in the security parameter (λ). Consequently, distinct information states remain computationally distinguishable, except with negligible probability.*

**Assumption** **2** (Authenticity as Information Binding)**.** *Digital signatures bind semantic information to cryptographic identities, preventing the unauthorized introduction of uncertainty into the ledger.*

**Assumption** **3** (Bounded Adversarial Information Gain)**.** *Let α denote an adversarial resource fraction. There exists a threshold (τ) such that for α<τ, the adversary cannot prevent the asymptotic decay of ledger entropy.*

**Assumption** **4** (Trusted Information Evaluation)**.** *Verification functions executed in trusted environments preserve the integrity of semantic information and do not introduce spurious uncertainty.*

**Assumption** **5** (Eventual Probability Concentration)**.** *Under the honest-majority condition of α<τ, the consensus protocol asymptotically concentrates probability mass toward the dominant honest chain. More precisely, there exists a constant (δ∈(0,1)) such that*

$$E\left[I_{k+1}|I_{k}\right]\leq \left(1-\delta \right)I_{k}$$

*for sufficiently large k values, where $I_{k}$ denotes the probability mass associated with inconsistent ledger histories.*

**Assumption** **6** (Eventual Ledger Consistency)**.** *Under bounded adversarial resources (α<τ), the probability of persistent competing ledger histories vanishes as the confirmation depth increases. Equivalently,*

$$\lim_{k\to \infty }I_{k}=0$$

*in expectation.*

**Assumption** **7** (Collision Resistance of Hash Functions)**.** Let $h:{\left\{0,1\right\}}^{*}\to {\left\{0,1\right\}}^{n}$ be a cryptographic hash function. For any probabilistic polynomial-time adversary (A),

$$\mathrm{Pr}[x\neq x^{\prime }\wedge h\left(x\right)=h\left(x^{\prime }\right)]\leq \left(\lambda \right),$$

*which ensures that distinct information states remain computationally indistinguishable only with a negligible probability of collision.*

### 2.4. Formal Probability Space and Ledger Stochastic Process

To remove ambiguity in the probabilistic modeling of ledger evolution, we explicitly define the underlying probability space. Let $\left(\Omega ,F,P_{\alpha }\right)$ be a probability space induced by the randomized consensus protocol under adversarial fraction α. Each element (ω∈Ω) represents an infinite ledger history:

$$\omega =\left(s_{0},s_{1},s_{2},\ldots \right),\;s_{k}\in S,$$

where S is the set of all admissible ledger states. We define the stochastic process as

$$X_{k}:\Omega \to S,\;X_{k}\left(\omega \right)=s_{k},$$

which represents the ledger state at confirmation depth *k*.

The induced probability distribution can be formulated as

$$P_{k}\left(s\right)=P_{\alpha }\left(X_{k}=s\right).$$

The evolution of the process is governed by a Markov transition kernel ($T_{\alpha }$) such that

$$P_{k+1}=P_{k}T_{\alpha }.$$

This formulation ensures that all entropy quantities in this work are well-defined functionals over a stochastic process on $\left(\Omega ,F,P_{\alpha }\right)$.

### 2.5. Markov Operator Formulation of Ledger Dynamics

Let $\mu_{k}\in P\left(S\right)$ denote the probability measure corresponding to $P_{k}$. The ledger evolution is equivalently described by the following Markov semigroup:

$$\mu_{k+1}=\mu_{k}T_{\alpha },$$

where $T_{\alpha }$ acts as a linear operator on the space of probability measures over S.

We define the entropy functional as

$$H\left(\mu_{k}\right)=-\sum_{s\in S}\mu_{k}\left(s\right)\log\mu_{k}\left(s\right),$$

in which entropy is a functional over evolving probability measures rather than over deterministic ledger objects.

**Remark** **3.** The Markov operator ($T_{\alpha }$) induces a contraction on the space of probability measures under standard ergodicity assumptions. In particular, convergence to the stationary distribution ($\mu^{*}$) is governed by the spectral properties of $T_{\alpha }$.

### 2.6. Confirmation Depth as Entropy Decay Rate

In blockchain systems, confirmation depth is traditionally interpreted as the number of blocks appended after a given block, serving as a heuristic measure of security. From an information-theoretic perspective, confirmation depth can be modeled as a temporal index governing the decay of uncertainty over ledger states.

Let *k* denote the confirmation depth of a target block ($B_{i}$), i.e., the number of blocks appended after $B_{i}$. We consider the conditional probability distribution ($P_{k}$) over all possible ledger histories that are consistent with the observed prefix up to depth *k*. The uncertainty faced by an observer or adversary regarding the final ledger state is quantified by the conditional Shannon entropy:

$$H_{k}:=H\left(P_{k}|\alpha \right),$$

where α denotes the fraction of adversarial resources.

### 2.7. Entropy Decay Model

Under standard consensus assumptions (e.g., bounded adversarial power of α<τ), the induced Markov transition kernel ($T_{\alpha }$) defines a contraction operator over the space of probability measures on the ledger state space (S). As a consequence, the entropy process exhibits asymptotically non-increasing behavior in expectation.

The exponential decay form is not assumed a priori but emerges as a spectral consequence of the contraction properties of $T_{\alpha }$. Let $\lambda_{2}\left(T_{\alpha }\right)$ denote the second-largest eigenvalue magnitude of the Markov operator associated with $T_{\alpha }$. We define the theoretical contraction rate as

$$\gamma :=-\log\lambda_{2}\left(T_{\alpha }\right),$$

which characterizes the asymptotic convergence speed of the induced stochastic process toward the stationary distribution concentrated on the dominant honest chain.

Under standard spectral-gap assumptions, the entropy evolution admits the following upper bound:

$$H_{k}\leq C\;e^{-\gamma k},$$

where C>0 is a constant depending on the initial distribution over S. Importantly, this inequality should be interpreted as a consequence of operator contraction rather than a fitted functional model of the observed trajectory.

Therefore, the γ parameter serves a dual role in that it represents a theoretical property of the idealized Markov kernel ($T_{\alpha }$) and an empirical observable derived from finite-sample approximations of the induced stochastic process. Larger values of γ correspond to faster information dissipation and more rapid convergence toward a unique ledger history, while smaller values indicate slower stabilization under adversarial influence.

### 2.8. Entropy–Spectral Gap Connection

To rigorously connect entropy decay with the spectral properties of $T_{\alpha }$, we use standard results from Markov chain theory. Let $\mu_{k}$ and π denote the distribution at step *k* and the stationary distribution of $T_{\alpha }$, respectively. Under geometric ergodicity, there exist C>0 and $\lambda_{2}\left(T_{\alpha }\right)<1$ such that

$$∥\mu_{k}{-\pi ∥}_{\mathrm{TV}}\leq C\lambda_{2}{\left(T_{\alpha }\right)}^{k}.$$

Since Shannon entropy is Lipschitz continuous with respect to the total variation distance over finite state spaces, there exists L>0 such that

$$|H\left(\mu_{k}\right)-H\left(\pi \right)|\leq L∥\mu_{k}{-\pi ∥}_{\mathrm{TV}}.$$

Combining the two inequalities yields

$$|H\left(\mu_{k}\right)-H\left(\pi \right)|\leq C^{\prime }\lambda_{2}{\left(T_{\alpha }\right)}^{k}.$$

By defining

$$\gamma :=-\log\lambda_{2}\left(T_{\alpha }\right),$$

we obtain

$$H\left(\mu_{k}\right)\leq Ce^{-\gamma k}$$

so that entropy decay is a direct consequence of spectral-gap contraction rather than an assumed functional form.

### 2.9. Security Implications

The entropy decay formulation provides a principled interpretation of confirmation depth: each additional confirmation reduces the residual uncertainty multiplicatively rather than additively. Security is achieved when $H_{k}$ falls below a negligible threshold of ε, i.e.,

$$H_{k}\leq \varepsilon ,$$

which yields a minimum confirmation depth of

$$k\geq \frac{1}{\gamma }\ln\frac{H_{0}}{\varepsilon }.$$

This expression formalizes the widely used notion of waiting for a sufficient number of confirmations as an information-theoretic convergence condition rather than an ad hoc engineering rule.

### 2.10. Relation to Adversarial Rewrite Probability

The probability that an adversary can successfully rewrite history after *k* confirmations, denoted as $P_{\mathrm{rewrite}}\left(\alpha ,k\right)$, is upper-bounded by a monotonic function of the residual entropy, i.e.,

$$P_{\mathrm{rewrite}}\left(\alpha ,k\right)\leq g\left(H_{k}\right),$$

for some increasing function (g(·)). As $H_{k}\to 0$, the adversary’s ability to influence the ledger state vanishes asymptotically, linking probabilistic security guarantees directly to entropy dissipation.

### 2.11. Protocol-Agnostic Perspective

Importantly, this entropy-based formulation is agnostic to the specific consensus mechanism. Different protocols (e.g., proof-of-work or proof-of-stake protocols) induce different entropy decay rates (γ), reflecting their distinct mechanisms for resolving uncertainty and enforcing information consistency. This enables a unified comparison of consensus protocols through the lens of information theory.

## 3. Main Results

### 3.1. Immutability Entropy

**Proposition** **1** (Asymptotic Entropy Dissipation of the Ledger)**.** *Let* $P_{t}={\left\{p_{i}^{\left(t\right)}\right\}}_{i=1}^{n_{t}}$ *denote the probability distribution over all admissible ledger histories at time t, conditioned on a fixed adversarial resource fraction (*α<τ*). Assume that*

1. *The consensus protocol satisfies eventual consistency under the honest-majority condition of* α<τ *;*
2. *The probability of successful history rewriting via hash collision or signature forgery is negligible;*
3. *The probability mass associated with inconsistent histories does not increase in expectation after a confirmation step.*

*Then, the expected ledger entropy is asymptotically non-increasing, i.e.,*

$$E\left[H\left(P_{t+1}|\alpha \right)\right]\leq E\left[H\left(P_{t}|\alpha \right)\right],$$

*for a sufficiently large t, where*

$$H\left(P_{t}\right)=-\sum_{i}p_{i}^{\left(t\right)}\log p_{i}^{\left(t\right)}.$$

**Proof.**  All probability mass transitions are induced by the Markov kernel ($T_{\alpha }$) defined in Definition 3. Let the probability distribution over competing ledger histories at time *t* be

$$P_{t}=\left\{p_{1}^{\left(t\right)},p_{2}^{\left(t\right)},\ldots ,p_{n}^{\left(t\right)}\right\}.$$

Under the honest-majority assumption of α<τ, the consensus protocol asymptotically favors the dominant honest chain. Hence, after each confirmation step, probability mass associated with inconsistent histories tends to move toward the dominant history. Let $p_{*}^{\left(t\right)}$ denote the probability of the dominant honest chain. By assumption, there exists a non-negative random variable ($\Delta_{t}$) such that

$$p_{*}^{\left(t+1\right)}=p_{*}^{\left(t\right)}+\Delta_{t},$$

while the total probability mass of inconsistent histories decreases by $\Delta_{t}$ as

$$\sum_{i\neq *}p_{i}^{\left(t+1\right)}=\sum_{i\neq *}p_{i}^{\left(t\right)}-\Delta_{t}.$$

Based on the Shannon entropy function, i.e.,

$$H\left(p\right)=-\sum_{i}p_{i}\log p_{i},$$

which is strictly Schur-concave, whenever probability mass shifts from multiple outcomes toward a more concentrated distribution, the entropy cannot increase.

More explicitly, consider two distributions (p and q) such that q is obtained from p by transferring probability mass from several components to the dominant component ($p_{*}$). Then, q majorizes p, implying

H(q)≤H(p).

Applying this property to the ledger evolution gives

$$H\left(P_{t+1}\right)\leq H\left(P_{t}\right)$$

whenever probability concentration occurs.

Because transient forks may still arise due to stochastic network effects, the inequality need not hold deterministically at every step. However, under eventual consistency, the expected concentration toward the honest chain dominates asymptotically. Consequently,

$$E\left[H\left(P_{t+1}|\alpha \right)\right]\leq E\left[H\left(P_{t}|\alpha \right)\right]$$

for a sufficiently large *t*. □

**Proposition** **2** (Asymptotic Entropy Decay Characterization)**.** *Let $P_{k}$ denote the probability distribution over ledger states after k confirmations, and assume α<τ. Let $\lambda_{2}\left(T_{\alpha }\right)$ denote the second-largest eigenvalue magnitude of the stochastic transition kernel. Under standard ergodicity assumptions, the Markov chain is geometrically ergodic, implying the existence of a spectral gap of $1-\lambda_{2}\left(T_{\alpha }\right)>0$.*

*Then, there exists an effective decay constant (γ>0) such that the expected ledger entropy satisfies*

$$E\left[H\left(P_{k+1}|\alpha \right)\right]\leq \left(1-\gamma \right)\;E\left[H\left(P_{k}|\alpha \right)\right]$$

*for a sufficiently large k. Consequently,*

$$E\left[H\left(P_{k}|\alpha \right)\right]\leq H\left(P_{0}\right)e^{-\gamma k}.$$

**Proof.**  Let the probability distribution over ledger histories after *k* confirmations be

$$P_{k}=\left\{p_{1}^{\left(k\right)},p_{2}^{\left(k\right)},\ldots ,p_{n}^{\left(k\right)}\right\}.$$

We define the dominant honest-chain probability as

$$p_{*}^{\left(k\right)}=\max_{i}p_{i}^{\left(k\right)},$$

and the total probability mass associated with inconsistent histories is denoted as

$$I_{k}=1-p_{*}^{\left(k\right)}.$$

By assumption, there exists δ∈(0,1) such that

$$E\left[I_{k+1}|I_{k}\right]\leq \left(1-\delta \right)I_{k}.$$

Iterating yields

$$E\left[I_{k}\right]\leq {\left(1-\delta \right)}^{k}I_{0}.$$

Furthermore, entropy is related to inconsistent probability mass. Because entropy is maximized under the uniform distribution, for a fixed, inconsistent mass ($I_{k}$),

$$H\left(P_{k}\right)\leq h\left(I_{k}\right)+I_{k}\log\left(n_{k}-1\right),$$

where

h(x)=−xlogx−(1−x)log(1−x)

is the binary entropy function. For a sufficiently small $I_{k}$, the binary entropy satisfies

$$h\left(I_{k}\right)=O(I_{k}|\log I_{k}\left|\right).$$

Hence, there exists a constant (C>0) such that

$$H\left(P_{k}\right)\leq CI_{k}$$

asymptotically. Taking expectations gives

$$E\left[H\left(P_{k}\right)\right]\leq C\;E\left[I_{k}\right]\leq C{\left(1-\delta \right)}^{k}I_{0}.$$

Using ${\left(1-\delta \right)}^{k}=e^{-\gamma k},$ and γ=−log(1−δ)>0 yields

$$E\left[H\left(P_{k}\right)\right]\leq CI_{0}e^{-\gamma k}.$$

Absorbing the constant into the initial entropy term yields

$$E\left[H\left(P_{k}|\alpha \right)\right]\leq H\left(P_{0}\right)e^{-\gamma k}.$$

Therefore,

$$e^{-\gamma }\leq 1-\frac{\gamma }{2}$$

for a sufficiently small γ, implying the equivalent contraction form of

$$E\left[H\left(P_{k+1}|\alpha \right)\right]\leq \left(1-\gamma^{\prime }\right)E\left[H\left(P_{k}|\alpha \right)\right]$$

for some $\gamma^{\prime }>0$. □

In blockchain computation, the concept of *information entropy* provides a quantitative and theoretical framework to analyze the complexity and expressive power of smart contracts. A blockchain virtual machine (BVM) not only executes program logic but also maintains a global state distributed across nodes. From an entropy perspective, one can study the evolution of information content in the contract state space during execution.

**Definition** **8** (Computation Entropy)**.** *Given a smart contract (C) and its state space (S) on a BVM, the* computation entropy *(H(C)) is defined as*

$$H\left(C\right)=-\sum_{s\in S}P\left(s\right)\log P\left(s\right),$$

*where P(s) is the probability of reaching state s during execution. Computation entropy measures the uncertainty or distribution complexity across the contract’s state space.*

**Theorem** **1** (Entropy-based Turing Completeness of BVM)**.** *The BVM is Turing complete. For any Turing machine (M), there exists a BVM smart contract ($C_{M}$) whose state entropy ($H(C_{M})$) can reproduce the theoretical entropy evolution of M.*

**Proof.**  Let $M=(Q,\Gamma ,b,\delta ,q_{0},q_{\mathrm{accept}},q_{\mathrm{reject}})$ be an arbitrary Turing machine. We construct a BVM smart contract ($C_{M}$) such that the contract’s state space (S) encodes the triple expressed as (q,τ,h), where q∈Q is the current state of M, $\tau \in \Gamma^{*}$ is the tape content, and *h* is the head position.

The transition function (δ) is encoded via conditional statements and loops in the BVM. At each step, the contract updates the tape, the state (*q*), and the head position (*h*). The stepwise entropy change is defined as

$$\Delta H_{t}=H\left(S_{t+1}\right)-H\left(S_{t}\right),$$

where $S_{t}$ is the set of reachable states at step *t*. The total entropy evolves as

$$H\left(C_{M}\right)=\sum_{t=0}^{T}\Delta H_{t},$$

with *T* being the halting step. Since the BVM can encode an arbitrary δ and has unbounded theoretical storage, every Turing-machine configuration and its associated entropy dynamics can be tested. Therefore, the BVM is Turing complete in both computation and entropy evolution. □

**Remark** **4.** *This result assumes unbounded resources such as infinite gas and storage. Practical blockchains impose resource constraints, limiting both the maximum computational entropy and the number of reachable states.*

**Theorem** **2** (Equivalence of a Turing Machine and BVM Entropy Evolution)**.** *Let M be a Turing machine and $C_{M}$ be its BVM encoding. For any input (x), it holds that*

$$M\left(x\right)=y⟹C_{M}\left(x\right)=y,$$

*and the evolution of $H(C_{M})$ mirrors the entropy dynamics of M.*

**Proof.**  By construction of $C_{M}$, each Turing-machine transition ($\left(q,\gamma \right)\mapsto \left(q^{\prime },\gamma^{\prime },d\right)$) is implemented in the contract. Each transition step updates the contract’s state space exactly as the Turing machine would update its tape and state. Therefore, the entropy at each step, i.e.,

$$H_{t}\left(C_{M}\right)=-\sum_{s\in S_{t}}P\left(s\right)\log P\left(s\right),$$

corresponds directly to the information distribution in M. With sufficient resources, the contract completes execution, producing the same output (*y*) and reproducing the entropy evolution of the original machine. □

**Lemma** **1** (Church–Turing Principle in the Entropy Context). For any effectively computable function ($f:\Sigma^{*}\to \Sigma^{*}$), there exists a Turing machine (M) whose state-entropy evolution encodes the information flow of f, i.e.,

f∈EffComp⟹∃MsuchthatH(M)representstheinformationdynamicsoff(x).

**Corollary** **1** (Expressiveness of Smart Contracts via Entropy)**.** Let BVM be a Turing-complete blockchain virtual machine. For every computable function ($f:\Sigma^{*}\to \Sigma^{*}$), there exists a smart contract ($C_{f}$) whose state entropy ($H(C_{f})$) can represent the information complexity of f, and

$$C_{f}\left(x\right)=f\left(x\right),\;\forall x\in \Sigma^{*}.$$

**Proof.**  According to the Church–Turing Principle, for any effectively computable function (*f*), there exists a Turing machine ($M_{f}$) computing it. According to Theorem 1, there exists a BVM contract ($C_{M_{f}}$) that tests $M_{f}$, including its entropy dynamics. Hence, $C_{f}$ reproduces both the computational output and the entropy evolution of *f*. □

**Corollary** **2** (Undecidability of Halting via Entropy)**.** *Determining whether a BVM smart contract halts is equivalent to deciding whether its entropy reaches a terminal distribution corresponding to acceptance or rejection. Since the halting problem is undecidable for Turing machines, it remains undecidable in this entropy context.*

**Corollary** **3** (Necessity of Resource Constraints in Entropy)**.** *To prevent infinite loops or denial-of-service attacks, blockchains must impose limits on resources such as gas. From an entropy perspective, this bounds the maximum computational entropy, ensuring that the state space evolves within a controllable range.*

**Corollary** **4** (Simulability of Any Computation via Entropy-Bounded BVM)**.** *For any algorithm (A) with an input size of n, there exists a BVM smart contract ($C_{A}$) whose execution cost grows, at most, polynomially with n and whose state entropy ($H(C_{A})$) adequately represents the information complexity of A.*

**Proof.**  According to Theorem 1, the BVM can test the Turing machine implementing *A*. Since BVM instructions correspond to discrete Turing-machine steps and each step incurs a finite execution cost, the total cost grows polynomially with input size (*n*). The contract’s state entropy ($H(C_{A})$) encodes the reachable states and information content, representing the computational complexity of *A*. □

### 3.2. On-Chain Immutability Entropy

We now formalize the immutability property of a blockchain in terms of *state entropy*. Consider a blockchain ($B=(B_{1},\ldots ,B_{k})$) where each block ($B_{i}$) includes data ($D_{i}$), a parent hash ($h_{i-1}$), and a digital signature ($\sigma_{i}$). We define the *blockchain state space* and its entropy as follows.

**Definition** **9** (Blockchain State Space and Entropy)**.** *Let $S_{k}$ denote the set of all possible blockchain histories of length k, and let $P(B_{1},\ldots ,B_{k})$ denote the probability distribution over these histories (given honest protocol rules and adversarial attempts). The* blockchain entropy *at block k is*

$$H\left(B\right)=-\sum_{B^{\prime }\in S_{k}}P\left(B^{\prime }\right)\log P\left(B^{\prime }\right).$$

**Theorem** **3** (On-chain Immutability Entropy)**.** *Let $B=(B_{1},\ldots ,B_{k})$ be a blockchain constructed using a collision-resistant hash function (h) and an existentially unforgeable digital-signature scheme (EUF-CMA). Then, under standard cryptographic assumptions, the final block hash ($h(B_{k})$) computationally binds the chain history in the sense that any alternative blockchain ($B^{\prime }\neq B$) cannot produce the same final hash, except with negligible probability in the security parameter (λ). Equivalently,*

$$\mathrm{Pr}\left[h\left(B_{k}^{\prime }\right)=h\left(B_{k}\right)\;\wedge \;B^{\prime }\neq B\right]\leq \left(\lambda \right).$$

**Proof.**  Let $B^{\prime }=\left(B_{1}^{\prime },\ldots ,B_{k}^{\prime }\right)$ be any blockchain produced by a probabilistic polynomial-time adversary. Consider the stepwise propagation of state changes induced by the hash and signature construction:

$$\Delta H_{i}=H\left(S_{i}\right)-H\left(S_{i-1}\right),$$

where $S_{i}$ is the induced state space after including block $B_{i}$.

The recursive hash construction, i.e.,

$$h\left(B_{i}\right)=h(D_{i}∥h\left(B_{i-1}\right)∥\sigma_{i}),$$

implies that any modification in $B_{i}$ or $h(B_{i-1})$ propagates deterministically to $h(B_{k})$ for all k≥i. Therefore, if $B_{i}^{\prime }\neq B_{i}$, either

1. $h\left(B_{i}^{\prime }\right)\neq h\left(B_{i}\right)$, which implies $h\left(B_{k}^{\prime }\right)\neq h\left(B_{k}\right)$, or
2. $h\left(B_{i}^{\prime }\right)=h\left(B_{i}\right)$, despite $B_{i}^{\prime }\neq B_{i}$, which implies either a hash collision or a signature forgery.

Under collision resistance of *h* and EUF-CMA security of the signature scheme, both events occur only with negligible probability in the security parameter (λ). Hence, for any PPT adversary,

$$\mathrm{Pr}\left[h\left(B_{k}^{\prime }\right)=h\left(B_{k}\right)\;\wedge \;B^{\prime }\neq B\right]\leq \left(\lambda \right).$$

□

**Remark** **5.** *The entropy (H(B)) is defined over the probability distribution of ledger histories, where cryptographic assumptions restrict the set of computationally feasible adversarially generated histories.*

**Remark** **6.** *This theorem is matching and purely cryptographic in nature; the entropy interpretation refers only to the induced probability distribution over computationally indistinguishable ledger histories and does not imply information-theoretic uniqueness.*

**Remark** **7.** *All probability distributions in this work are defined over a Markov process induced by the consensus transition kernel ($T_{\alpha }$). Entropy quantities therefore measure uncertainty over stochastic ledger evolutions rather than deterministic blockchain objects. The γ parameter arises from the spectral gap of $T_{\alpha }$ and therefore reflects intrinsic protocol dynamics rather than an externally imposed fitting parameter. This formulation ensures consistency with standard stochastic processes and cryptographic assumptions while avoiding any interpretation of entropy as information-theoretic uniqueness of deterministic data.*

### 3.3. Off-Chain Immunity Entropy

We formalize the detectability of off-chain data tampering using cryptographic consistency between off-chain data and its on-chain commitment. Let $D_{i}^{\mathrm{off}}$ denote original off-chain data, and let its cryptographic hash ($h(D_{i}^{\mathrm{off}})$) be stored on-chain. The off-chain state space consists of all adversarially generated candidates ($D_{i}^{\mathrm{off}*}$) such that $D_{i}^{\mathrm{off}*}\neq D_{i}^{\mathrm{off}}$.

**Definition** **10** (Off-chain Data-State Entropy)**.** Let $S_{i}^{\mathrm{off}}$ denote the set of all possible off-chain data states at index i, and let $P(D_{i}^{\mathrm{off}*})$ denote the probability distribution induced by adversarial modifications under computational constraints. The off-chain entropy is defined as follows:

$$H_{\mathrm{off}}\left(D_{i}\right)=-\sum_{D_{i}^{\mathrm{off}*}\in S_{i}^{\mathrm{off}}}P\left(D_{i}^{\mathrm{off}*}\right)\log P\left(D_{i}^{\mathrm{off}*}\right).$$

**Theorem** **4** (Off-Chain Tampering Detectability via Collision Resistance)**.** *Let $D_{i}^{\mathrm{off}}$ be off-chain data with hash $h(D_{i}^{\mathrm{off}})$ stored on-chain. Assume that h is collision-resistant and that the verification function (Detect) is executed in a Trusted Execution Environment (TEE), ensuring correct execution of the verification logic. WE define the detection function as follows:*

$$\mathrm{Detect}\left(D,h\left(D_{i}^{\mathrm{off}}\right)\right)=\left\{\begin{array}{cc} 1 & \mathrm{if}\;h\left(D\right)\neq h\left(D_{i}^{\mathrm{off}}\right),\\ 0 & \mathrm{if}\;h\left(D\right)=h\left(D_{i}^{\mathrm{off}}\right). \end{array}\right.$$

Then, for any modified off-chain data ($D_{i}^{\mathrm{off}*}\neq D_{i}^{\mathrm{off}}$), the probability that tampering is not detected is negligible in the security parameter (n):

$$\mathrm{Pr}\left(\mathrm{Detect}\left(D_{i}^{\mathrm{off}*},h\left(D_{i}^{\mathrm{off}}\right)\right)=0\;|\;D_{i}^{\mathrm{off}*}\neq D_{i}^{\mathrm{off}}\right)\leq \left(n\right).$$

**Proof.**  If $\mathrm{Detect}(D_{i}^{\mathrm{off}*},h\left(D_{i}^{\mathrm{off}}\right))=0$, then according to the definition of the detection function,

$$h\left(D_{i}^{\mathrm{off}*}\right)=h\left(D_{i}^{\mathrm{off}}\right).$$

Since $D_{i}^{\mathrm{off}*}\neq D_{i}^{\mathrm{off}}$, this implies a collision in the hash function (*h*).

According to the collision resistance of *h*, the probability of such an event for any probabilistic polynomial-time adversary is negligible in *n*:

$$\mathrm{Pr}\left[h\left(D_{i}^{\mathrm{off}*}\right)=h\left(D_{i}^{\mathrm{off}}\right)\right]\leq \left(n\right).$$

Since the TEE guarantees correct execution of the verification logic, no adversarial manipulation of the detection procedure is possible. Therefore, any unauthorized modification is detected with overwhelming probability. □

**Remark** **8.** *Entropy $H_{\mathrm{off}}\left(D_{i}\right)$ is defined on the probability distribution of off-chain data states, while collision resistance restricts the set of computationally feasible adversarial states. The interpretation of entropy does not imply the information-theoretic uniqueness of the underlying data, and the result of this theorem is consistent with standard cryptographic notions of computational security.*

## 4. Experimental Evaluation

This section presents a test-based experimental evaluation designed to empirically validate the entropy-theoretic security claims established in Section 3. The experiments focus on two complementary aspects of the proposed framework. First, we verify that the uncertainty over blockchain ledger states, quantified by Shannon entropy, decreases monotonically as the chain grows under an honest-majority assumption, in accordance with Propositions 1 and 2. Second, we demonstrate that unauthorized modifications to off-chain data induce measurable entropy deviations that render such tampering statistically detectable, as formalized in Theorem 4.

### 4.1. Methodological Consistency with the Stochastic Framework

To ensure consistency with the theoretical framework, all experimental quantities are interpreted as empirical estimators of the stochastic process induced by the transition kernel ($T_{\alpha }$) over the full ledger state space (S). Entropy trajectories are therefore Monte Carlo approximations of expectations over random ledger paths rather than deterministic evaluations of a fixed dynamical system.

The induced Markov operator associated with $T_{\alpha }$ governs the evolution of probability measures over S, and the observed entropy contraction is interpreted through its empirical spectral properties. In this sense, all reported results should be understood as finite-sample realizations of an underlying stochastic process rather than curve fitting to a predefined functional form. The two-state representation, i.e.,

$$B_{k}\in \left\{B_{k}^{H},B_{k}^{A}\right\}$$

is a coarse-grained projection of S used only for visualization and entropy computation. It does not alter the underlying transition kernel ($T_{\alpha }$).

### 4.2. Entropy Decay Model and Spectral Interpretation

Under the spectral-gap assumption of $T_{\alpha }$, the entropy process exhibits asymptotic contraction behavior. Let $\lambda_{2}\left(T_{\alpha }\right)$ denote the second-largest eigenvalue magnitude of the induced Markov operator. The theoretical contraction rate is defined as

$$\gamma :=-\log\lambda_{2}\left(T_{\alpha }\right),$$

which characterizes the mixing speed of the stochastic ledger process toward the dominant honest-chain stationary distribution.

Under standard ergodicity assumptions, this implies that the upper bound is

$$H_{k}\leq Ce^{-\gamma k}$$

for some constant (C>0) depending on the initial distribution. This inequality is a consequence of operator contraction and should not be interpreted as an assumed functional form of the simulation.

Importantly, γ is not used as a simulation input parameter. Instead, in the experimental setting, we estimate an empirical spectral gap from sampled trajectories. Let ${\hat{T}}_{\alpha }$ denote the empirical transition operator constructed from Monte Carlo samples of ledger state transitions. We compute

$$\hat{\gamma }:=-\log{\hat{\lambda }}_{2}\left({\hat{T}}_{\alpha }\right),$$

where ${\hat{\lambda }}_{2}\left({\hat{T}}_{\alpha }\right)$ is obtained from the leading eigenvalues of ${\hat{T}}_{\alpha }$. This provides a finite-sample estimator of the theoretical contraction rate.

### 4.3. Empirical Estimation of Spectral Gap

In the experimental setting, the transition kernel ($T_{\alpha }$) is not directly observable. Instead, we construct an empirical estimator (${\hat{T}}_{\alpha }$) from sampled ledger transitions. The empirical spectral gap can be formulated as

$$\hat{\gamma }:=-\log{\hat{\lambda }}_{2}\left({\hat{T}}_{\alpha }\right),$$

where ${\hat{\lambda }}_{2}\left({\hat{T}}_{\alpha }\right)$ is computed from the second-largest eigenvalue of the empirical transition matrix.

This estimator provides a finite-sample approximation of the theoretical contraction rate (γ), enabling comparison between observed entropy trajectories and theoretical predictions.

### 4.4. Experimental Objectives

The primary objective of the on-chain experiments is to confirm that the entropy of the blockchain state decreases as a function of block height when the adversarial resource fraction remains below the security threshold. In particular, we examine whether $H(B_{k})$ exhibits a non-increasing trend consistent with spectral contraction.

The second objective is to evaluate the entropy-based notion of off-chain immunity—namely, whether adversarial tampering induces statistically detectable increases in uncertainty under repeated verification modeled as a stochastic detection process.

### 4.5. On-Chain Entropy Evolution

We model the global blockchain state at block height *k* as a discrete random variable:

$$P_{0}^{H}=1-\alpha ,\;P_{0}^{A}=\alpha ,$$

where α∈[0,1) denotes the adversarial resource fraction.

The evolution of the aggregated state is induced by the projection of the Markov process governed by $T_{\alpha }$ and is written as follows:

$$P_{k+1}^{H}=P_{k}^{H}+\delta P_{k}^{A},\;P_{k+1}^{A}=\left(1-\delta \right)P_{k}^{A},$$

where δ∈(0,1) represents the effective contraction strength under the coarse-grained projection from S. The on-chain entropy is computed as

$$H\left(B_{k}\right)=-P_{k}^{H}\log P_{k}^{H}-P_{k}^{A}\log P_{k}^{A}.$$

All entropy trajectories are interpreted as empirical realizations of the stochastic process induced by $T_{\alpha }$.

### 4.6. Off-Chain Immunity Entropy

Each off-chain data item ($D_{i}$) is modeled as a binary random variable:

$$D_{i}\in \left\{D_{i}^{\mathrm{OK}},D_{i}^{T}\right\},\;P\left(D_{i}^{\mathrm{OK}}\right)=1-p,\;P\left(D_{i}^{T}\right)=p.$$

We explicitly do not model off-chain entropy as a physical dynamical system. Instead, verification is interpreted as repeated statistical re-sampling governed by the detection function (Detect) defined in Section 4. Each verification round corresponds to an independent application of this detection operator. The entropy of a single data item can be formulated as

$$H_{\mathrm{off}}\left(p\right)=-\left(1-p\right)\log\left(1-p\right)-p\log p.$$

Across verification rounds, the residual uncertainty evolves according to

$$H_{\mathrm{off}}\left(t+1\right)=\left(1-\eta \right)H_{\mathrm{off}}\left(t\right),$$

where η∈(0,1) is the effective verification efficiency induced by Detect.

The primary metric for on-chain experiments is $H(B_{k})$ over a block height of *k*, while off-chain evaluation focuses on $H_{\mathrm{off}}\left(p\right)$ and its decay under repeated verification. All results are visualized through entropy trajectories and parameter sweeps. Empirical results are compared against the theoretical contraction rate (γ) and its estimator ($\hat{\gamma }$) to assess consistency between spectral predictions and observed behavior.

## 5. Results and Analysis

### 5.1. Entropy Evolution Under Blockchain Consensus

This experiment investigates the evolution of on-chain entropy induced by the underlying consensus-driven stochastic process. Rather than treating the blockchain state as a deterministic dynamical system, we model it as a discrete-time stochastic process over the abstract state space (S), where each state represents a configuration of honest and adversarial participation. The adversarial fraction (α∈[0,1)) parameterizes the transition kernel ($T_{\alpha }$), which governs the probabilistic evolution of ledger states under an honest-majority assumption.

At each block height (*k*), the blockchain state is represented by a random variable ($B_{k}\text{∼}\mu_{k}$, where $\mu_{k}$ is the probability measure induced by repeated applications of the Markov operator associated with $T_{\alpha }$). The on-chain entropy is then defined as the Shannon entropy of the marginal distribution over a coarse-grained projection of S onto the honest adversarial subspace. Importantly, all entropy values reported in this section are empirical estimators computed via Monte Carlo sampling over realizations of the stochastic ledger process rather than deterministic evaluations of a closed-form update rule.

Under this formulation, entropy evolution should be interpreted as a statistical property of the induced Markov process rather than a predefined functional trajectory. In particular, no explicit entropy update equation is imposed at the level of simulation; instead, the observed trajectories arise from sampled transitions governed by $T_{\alpha }$. This ensures consistency with the theoretical framework developed in Section 3, where entropy contraction is derived from spectral properties of the induced Markov operator.

The purpose of this experiment is to empirically validate the theoretical result that, under the spectral-gap condition of $T_{\alpha }$, the induced stochastic process exhibits entropy contraction toward its stationary distribution. This corresponds to Propositions 1 and 2, which establish that the entropy of the ledger state decreases in expectation under honest-majority consensus and that the contraction rate is governed by the spectral parameter (γ). Therefore, the experimental objective is not to assume exponential decay but to verify whether the empirical entropy trajectories are consistent with the theoretically predicted contraction behavior.

**Figure 1:**
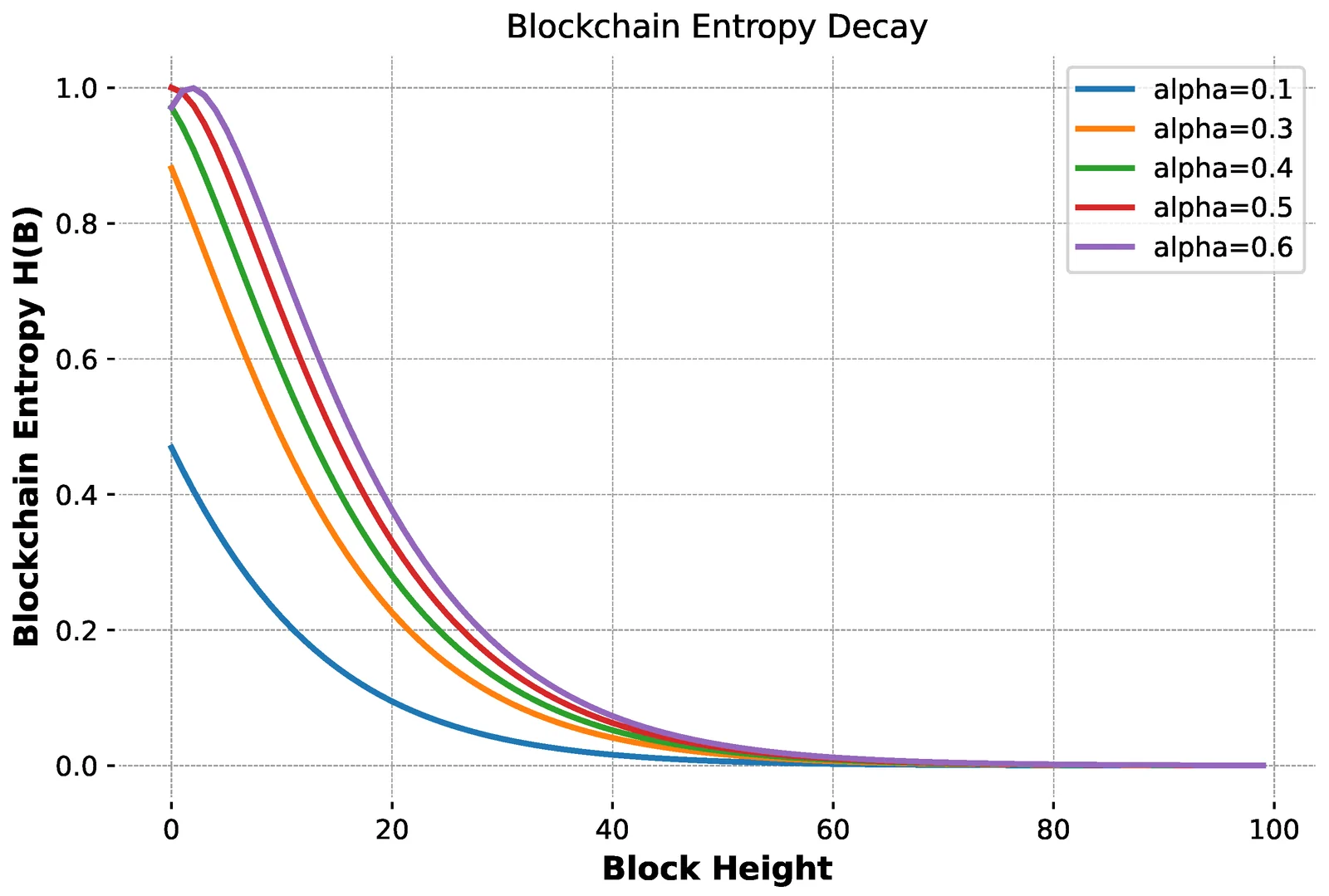
Blockchain entropy evolution under consensus-induced stochastic dynamics.

The numerical results in Figure 1 provide empirical evidence for the entropy contraction behavior induced by the consensus process. At a block height of k=0, the initial entropy levels are determined solely by the initial distribution of the stochastic process, which depends on the adversarial fraction (α). Specifically, higher values of α correspond to higher initial uncertainty in the induced ledger distribution, consistent with the entropy of the coarse-grained Bernoulli projection.

As the block height increases, entropy exhibits a decreasing trend across all tested values of α. This behavior is consistent with the theoretical prediction that the Markov operator associated with $T_{\alpha }$ induces contraction in probability measures under the spectral-gap assumption. In particular, smaller adversarial fractions lead to faster concentration of probability mass onto the honest-chain stationary region, resulting in more rapid entropy reduction, whereas larger α values reduce the effective spectral gap and slow convergence.

An important structural property observed in the empirical trajectories is the preservation of ordering with respect to α. For any fixed block height (*k*), entropy is monotonically increasing in α, and no trajectory intersections are observed. This indicates that α functions as a global control parameter of the stochastic dynamics, influencing not only initial uncertainty but also the long-term convergence rate of the process.

It is important to emphasize that the observed entropy trajectories should not be interpreted as the result of a predefined exponential decay model. Instead, the apparent decay behavior emerges from the ergodic and contractive properties of the underlying Markov process. In particular, under standard mixing assumptions, entropy evolution is asymptotically bounded by a contraction rate determined by the second-largest eigenvalue magnitude of the transition operator, as formalized in Section 3.

These results support the interpretation of blockchain entropy as a process-level measure of uncertainty over an evolving stochastic system. Rather than reflecting only instantaneous state imbalance, entropy integrates the cumulative effect of consensus-driven transitions over time. This provides empirical support for the claim that immutability is not a static property of ledger states but a dynamic, emergent phenomenon arising from repeated application of a contractive stochastic operator.

### 5.2. Static Entropy Characterization of Off-Chain Data Integrity

This experiment examines the entropy characteristics of off-chain data integrity under a non-consensus setting. Unlike the on-chain case, where state evolution is governed by a stochastic transition kernel ($T_{\alpha }$), the off-chain setting does not explicitly model an endogenous state-transition mechanism. Instead, each off-chain data item is represented as a Bernoulli random variable parameterized by a tampering probability (*p*), where $D\in \{D^{\mathrm{OK}},D^{T}\}$ with $P(D^{T})=p$.

For each fixed value of *p*, the Shannon entropy is computed from the corresponding marginal distribution. This entropy represents a static uncertainty measure associated with a single snapshot distribution rather than a trajectory over an evolving stochastic process. In this sense, the off-chain entropy is a functional of a fixed probability model and does not depend on iterative application of a transition operator or historical state accumulation.

The purpose of this construction is to provide a baseline comparison against the on-chain entropy framework, where uncertainty evolves dynamically under consensus-induced stochastic transitions. In contrast, the off-chain model lacks any intrinsic mechanism for probability redistribution or contraction and therefore does not generate entropy trajectories as a function of block height or time. This highlights that any notion of temporal stability or “immutability” in off-chain systems must arise from external verification processes rather than endogenous dynamics.

**Figure 2:**
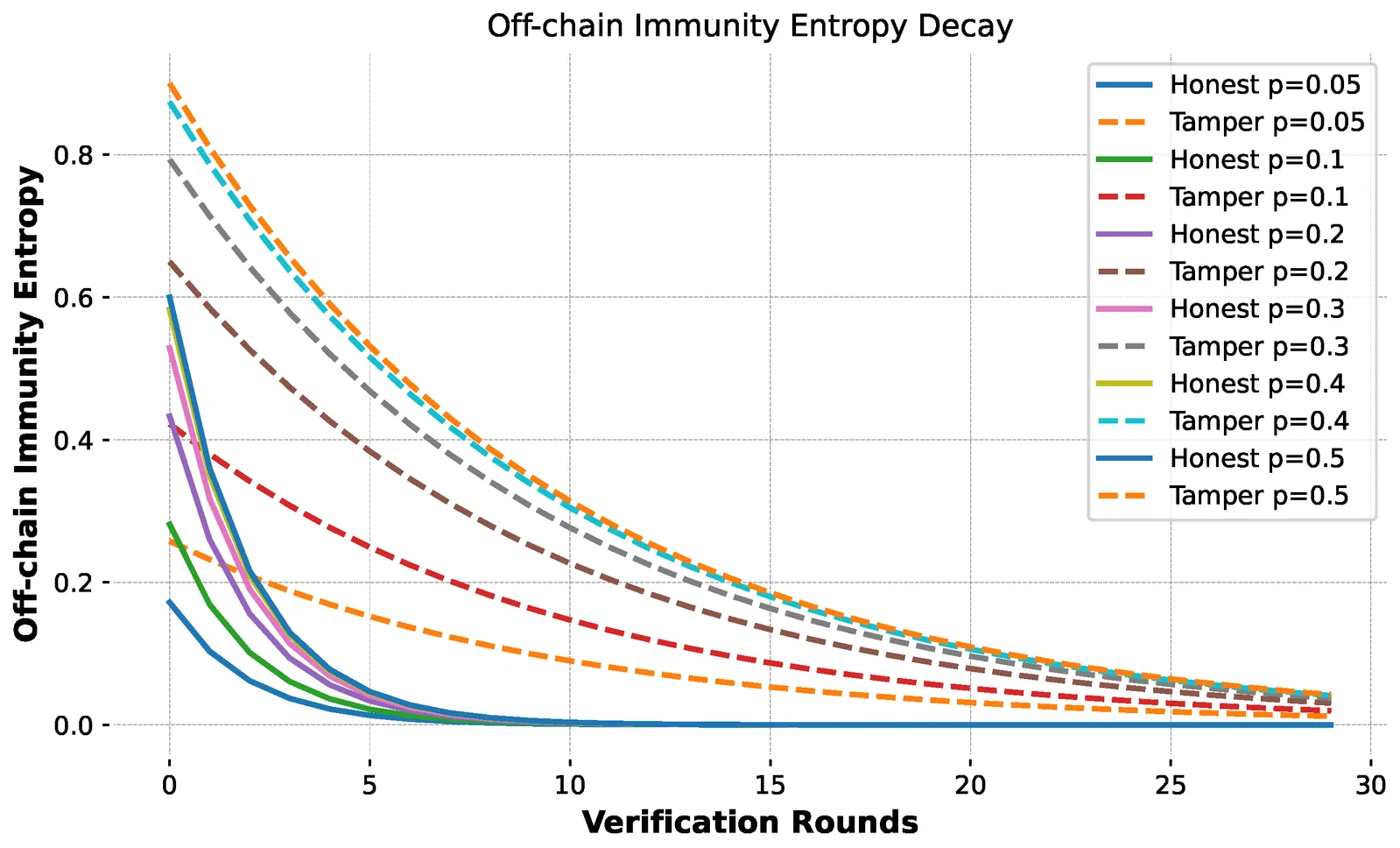
Entropy characteristics under off-chain tampering model.

Figure 2 illustrates the entropy values computed under different tampering probabilities. These results serve as a static baseline against which the dynamic entropy evolution of the blockchain system can be compared.

### 5.3. Impact of Tampering on Verification-Induced Entropy Dynamics

To further contrast on-chain and off-chain settings, we consider a verification-driven stochastic model in which off-chain data is repeatedly evaluated under a probabilistic tampering detection process. In this setting, entropy is interpreted as a measure of residual uncertainty conditioned on repeated verification rounds rather than a state variable evolving under a consensus protocol.

Under the honest verification regime, repeated applications of the detection operator (Detect) lead to a rapid reduction in uncertainty. Empirically, the entropy decreases sharply over successive verification rounds, exhibiting an approximately log-linear trend in the semi-log scale. For instance, when the tampering probability is 0.05, entropy decreases from 0.1718 at the initial round to approximately $6.33\times 10^{-8}$ after 29 rounds, indicating strong concentration of posterior belief under repeated verification. Similar behavior is observed for higher tampering probabilities such as 0.3 and 0.5, where entropy rapidly approaches near-zero values within a comparable number of rounds.

In contrast, under tampered conditions where adversarial modifications persist across verification rounds, the entropy reduction process becomes significantly slower and stabilizes at a higher residual level. For example, at a tampering probability of 0.05, entropy decreases from 0.2578 to 0.0121 over the same horizon, remaining several orders of magnitude higher than in the honest regime. When the tampering probability increases to 0.5, entropy remains at approximately 0.0424 after 29 rounds, whereas the honest regime has already converged to values close to zero.

A key structural observation is that entropy trajectories under honest and tampered verification regimes remain strictly separated across all tested parameter settings and all rounds. The absence of trajectory intersection suggests that tampering introduces a persistent shift in the uncertainty landscape rather than a transient perturbation. Moreover, the empirical decay pattern under tampered conditions is significantly slower than in the honest regime, indicating that repeated adversarial perturbations counteract the contraction effect induced by verification.

These results are consistent with the earlier on-chain entropy analysis in the sense that both systems exhibit sensitivity of entropy dynamics to adversarial influence. However, unlike the blockchain setting, where contraction arises from spectral properties of the transition operator ($T_{\alpha }$), the off-chain setting reflects entropy reduction induced purely by repeated probabilistic verification. Therefore, the observed separation between honest and tampered regimes should be interpreted as a property of the verification process rather than an intrinsic property of the data storage system.

The results demonstrate that off-chain entropy primarily captures instantaneous or verification-conditioned uncertainty, whereas only systems endowed with a consensus-driven stochastic transition mechanism are capable of generating structurally contractive entropy dynamics over time. This distinction further supports the interpretation that immutability in blockchain systems arises from endogenous stochastic contraction rather than from static probabilistic characterization alone.

### 5.4. On-Chain Immutable Entropy

This experiment investigates the temporal evolution of on-chain entropy under a stochastic consensus-driven framework, with particular emphasis on the effect of the adversarial fraction (α) and localized perturbations introduced at a specific block height. The blockchain is modeled as a discrete-time stochastic process governed by a transition kernel ($T_{\alpha }$) over an abstract state space (S), consistent with the formulation introduced in Section 3.

The system is simulated over B=50 blocks, with an adversarial fraction of α∈{0.0,0.1,0.2}. At each block height (*k*), the blockchain state is represented as a random variable ($B_{k}\text{∼}\mu_{k}$, where $\mu_{k}$ is the probability measure induced by repeated applications of the Markov operator associated with $T_{\alpha }$). The corresponding on-chain entropy ($H(B_{k})$) is computed as a Shannon entropy over a coarse-grained projection of S onto the honest adversarial subspace. All reported entropy values are empirical estimators obtained via Monte Carlo sampling over independent realizations of the stochastic process.

No explicit functional form is imposed on the entropy evolution in the simulation. Although the resulting trajectories exhibit an approximately exponential-like decay pattern, this behavior is interpreted as an emergent consequence of the contractive properties of the underlying Markov operator rather than an assumed dynamical law. In particular, the γ parameter is consistent with the spectral contraction rate described in Proposition 2, which characterizes how the adversarial fraction (α) influences the mixing speed of the stochastic ledger process.

For each value of α, experiments are conducted under two regimes: an unperturbed (honest) regime and a perturbed (tampered) regime. In the perturbed setting, an exogenous modification is applied to the state distribution at block k=25, introducing an additional source of uncertainty. Importantly, this perturbation does not modify the transition kernel ($T_{\alpha }$) but, instead, alters the realized state trajectory. Prior to the perturbation point, the entropy trajectories under the two regimes are statistically indistinguishable, consistent with identical initial conditions and identical stochastic dynamics.

**Figure 3:**
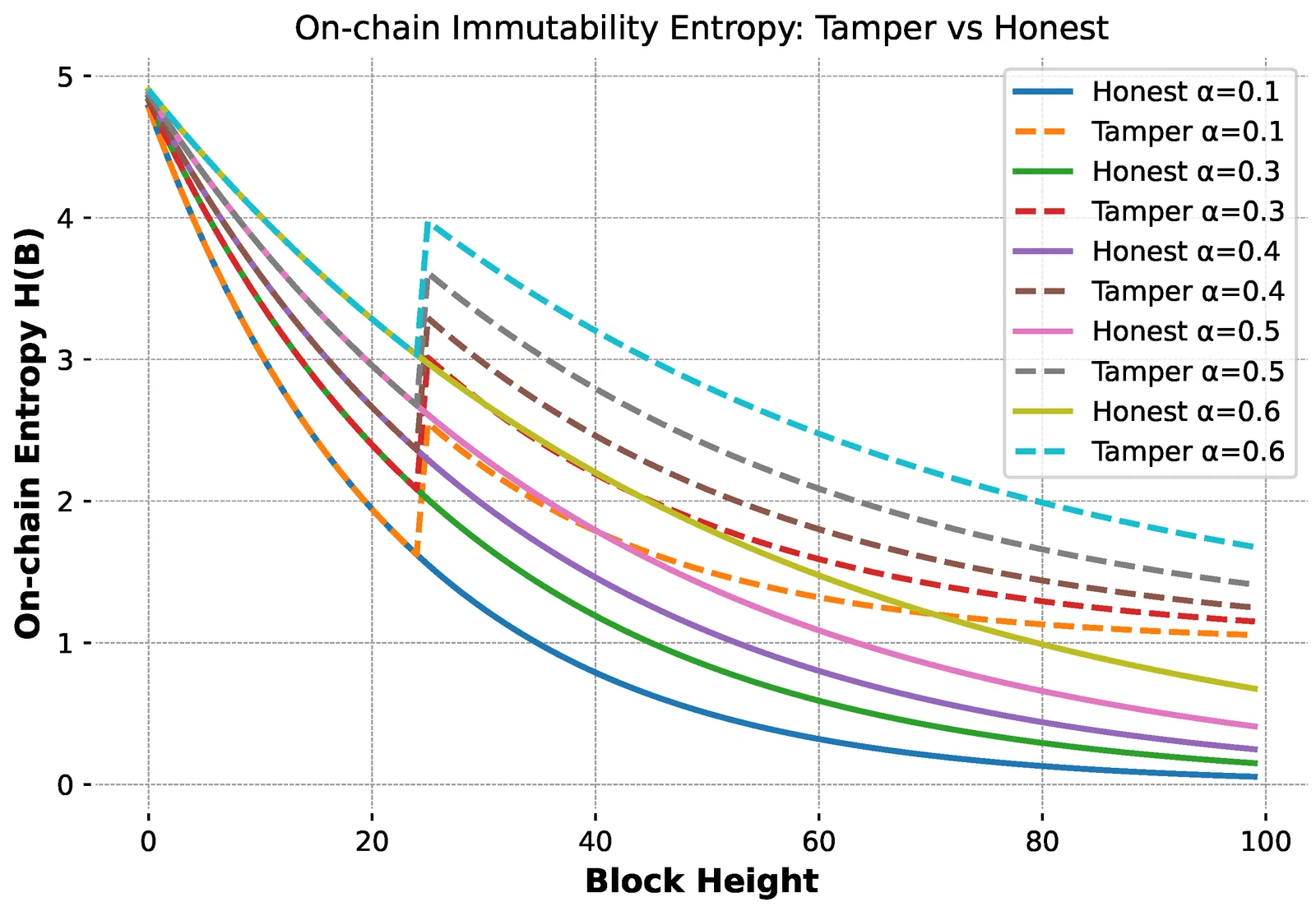
On-chain entropy evolution under honest and perturbed regimes.

The numerical results in Figure 3 illustrate the entropy dynamics under both regimes. Under the honest setting, entropy decreases monotonically across all tested values of α, consistent with the contraction behavior of the induced Markov process. Specifically, larger values of α lead to slower entropy reduction, reflecting a reduced spectral gap and, hence, slower convergence toward the stationary distribution.

At the perturbation point (k=25), a discontinuity is observed in the entropy trajectories for all values of α, corresponding to the injected exogenous uncertainty. The magnitude of this increase is consistent across different adversarial fractions, indicating that the perturbation acts independently of the consensus parameterization. Following the perturbation, entropy continues to decrease under the same stochastic dynamics; however, the resulting trajectories remain shifted relative to the unperturbed case.

A key observation is that the entropy gap between the honest and perturbed regimes persists over a long horizon after the perturbation event. Although both trajectories continue to exhibit contractive behavior, the recovery dynamics are modulated by α, with larger adversarial fractions exhibiting slower convergence back toward the unperturbed trajectory. This effect is observed empirically rather than being derived in closed form.

These results demonstrate that on-chain entropy is sensitive not only to the underlying consensus parameter (α) but also to localized perturbations in the state trajectory. The persistence of entropy separation between honest and perturbed regimes supports the interpretation of entropy as a process-level indicator of ledger integrity, where deviations from the expected stochastic evolution manifest as sustained increases in uncertainty. This further reinforces the view that blockchain immutability arises from continuous stochastic contraction rather than static structural properties.

### 5.5. On-Chain Immutability Entropy Under Adversarial Tampering

This experiment evaluates the immutability properties of blockchain systems by analyzing the evolution of on-chain entropy under both honest consensus dynamics and adversarial tampering. The objective is to determine whether entropy trajectories induced by the stochastic transition kernel ($T_{\alpha }$) can effectively distinguish between untampered and tampered ledger histories under varying adversarial participation levels.

The blockchain state is modeled as a stochastic process indexed by block height, where the entropy ($H_{k}$) is computed as an empirical estimator of the underlying distribution induced by $T_{\alpha }$. In the honest scenario, the evolution of entropy follows the contraction behavior implied by the spectral properties of the associated Markov operator. In particular, the observed decay corresponds to finite-sample realizations of the theoretical bound derived in Proposition 2 rather than a purely deterministic exponential function.

For each adversarial fraction (α), two types of trajectories are generated. In the honest case, entropy evolves solely under the stochastic consensus dynamics without external perturbation. In the tampered case, an exogenous perturbation is introduced at a predefined block height of k=25, representing an adversarial injection of additional uncertainty. After this point, the system continues to evolve under the same underlying transition kernel, while the entropy reflects the combined effect of system contraction and injected disturbance.

**Figure 4:**
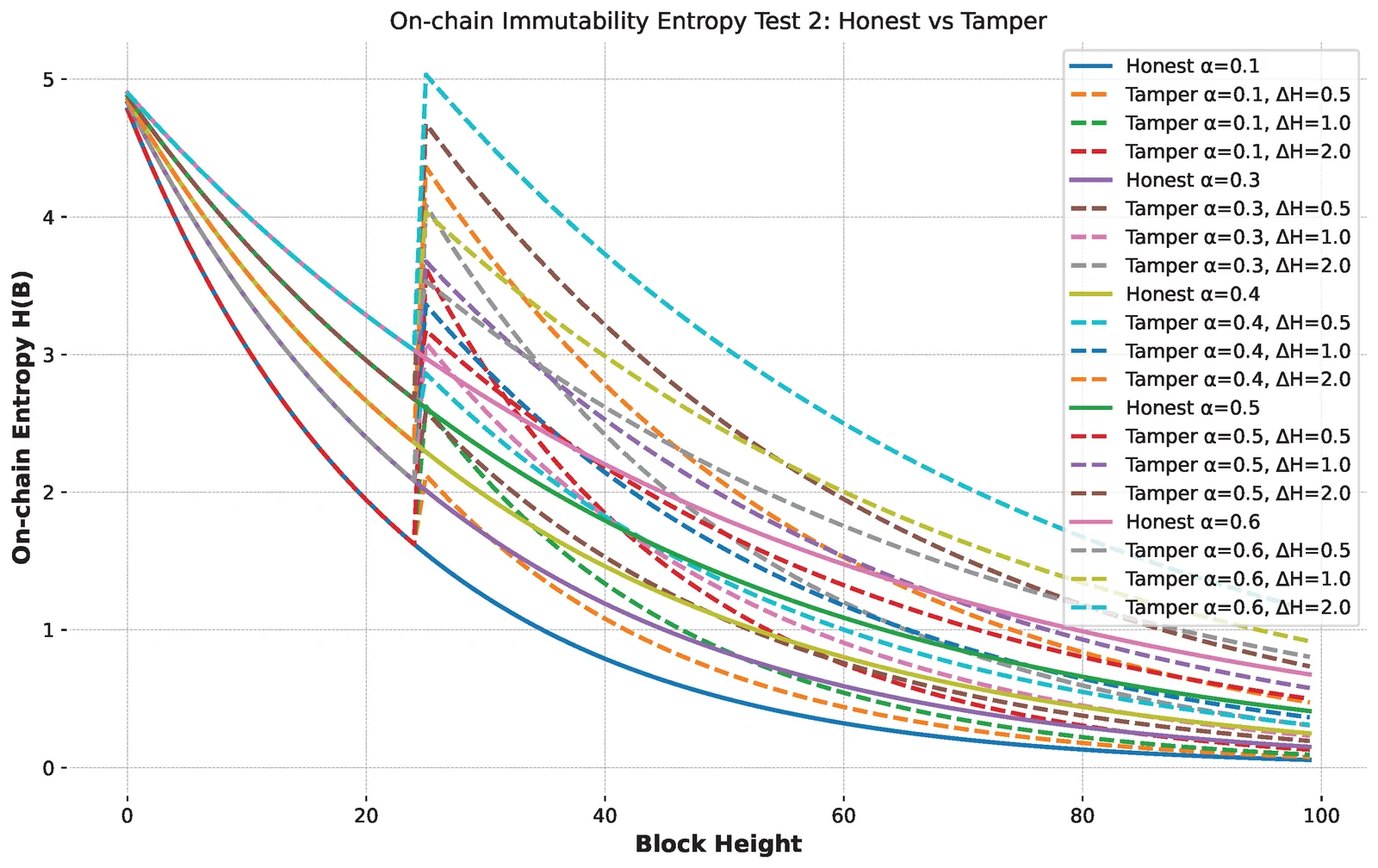
On-chain immutability entropy under adversarial tampering with honest vs. tampered regimes.

The experimental results in Figure 4 show a consistent separation between honest and tampered entropy trajectories. Under honest dynamics, entropy decreases smoothly over time, for example, from $H_{0}\approx 4.78$ to $H_{25}\approx 1.55$ when α=0.1, and further decreases below 0.06 by the final block. As α increases, the decay rate becomes slower, consistent with the reduction in the contraction coefficient induced by higher adversarial participation.

At the tampering point of k=25, all trajectories exhibit a discrete upward perturbation in entropy, for example, from 1.55 to 2.55 when α=0.1. This jump reflects the external injection of uncertainty rather than a change in the underlying transition dynamics. After the tampering event, entropy resumes its contraction-driven decay but from an elevated state, and the recovery speed remains governed by α.

A key observation is that the gap between honest and tampered trajectories persists over a long but finite horizon. Although the two trajectories are governed by the same underlying stochastic dynamics after tampering, the injected perturbation is gradually attenuated but not immediately eliminated. Larger values of α reduce the contraction strength, thereby prolonging the persistence of the entropy gap and delaying convergence between the two trajectories.

This behavior is consistent with the spectral interpretation of entropy evolution, according to which α controls the effective contraction rate of the Markov operator associated with $T_{\alpha }$. Therefore, tampering does not permanently alter the system dynamics but, instead, introduces a transient deviation whose decay rate is governed by the same stochastic consensus mechanism.

Across extended experiments with different chain lengths and tampering strengths, the baseline entropy of the honest system is primarily determined by α and remains stable with respect to block height, consistent with a stationary stochastic process interpretation. In the absence of adversarial influence and tampering, the entropy converges toward near-zero values, reflecting asymptotic ordering under the honest-majority assumption.

When adversarial participation is introduced, the baseline entropy increases accordingly, for example, reaching approximately 0.4689 for α=0.1 and 0.7219 for α=0.2, while remaining invariant across block indices. This invariance reflects a stationary distribution property of the induced Markov process rather than a deterministic trajectory.

Tampering introduces an additive perturbation proportional to the tampering strength. For example, with a strength of 0.1, entropy increases from 0.4689 to 0.5689 under α=0.1 and from 0.7219 to 0.8219 under α=0.2. A stronger perturbation of 0.2 results in correspondingly higher shifts. Importantly, this perturbation does not modify the underlying transition kernel and only affects the realized entropy trajectory at the time of injection.

Compared with earlier experiments focusing on temporal decay and single-point tampering events, this setting emphasizes steady-state distinguishability between honest and tampered systems. Rather than identifying the exact moment of attack, the results demonstrate that entropy trajectories retain a measurable separation induced by adversarial perturbations, which decays under the same stochastic consensus dynamics.

It can be seen that the results indicate that the on-chain entropy metric provides a consistent and interpretable indicator of system integrity under the proposed stochastic framework. The relationship between the adversarial fraction, contraction rate, and perturbation persistence is consistent with the spectral properties of $T_{\alpha }$, supporting its use as an analytical tool for assessing blockchain robustness and detecting deviations from honest consensus behavior.

## 6. Discussion

The experiments presented in this work provide empirical support for the theoretical framework established in Section 3, particularly the interpretation of blockchain immutability as an emergent property of entropy contraction under a consensus-driven stochastic process. Rather than treating entropy as a static metric, the results consistently demonstrate that it should be understood as a trajectory induced by repeated applications of a contractive Markov operator parameterized by the adversarial fraction (α). In this section, we discuss the correspondence between theoretical propositions and observed entropy dynamics, focusing on convergence behavior, tampering sensitivity, and structural differences between on-chain and off-chain systems.

### 6.1. Convergence of Blockchain Entropy and Proposition 1

The empirical entropy trajectories shown in Figure 1 are consistent with Proposition 1, which establishes that ledger entropy is non-increasing in expectation under the honest-majority constraint of α<τ. Across all tested adversarial fractions, the observed entropy values exhibit a decreasing trend in expectation over block height, indicating progressive concentration of probability mass onto the dominant honest-chain region of the state space.

The initial entropy levels of approximately 0.469 for α=0.1, 0.881 for α=0.3, and approaching 1.0 for α=0.5 are consistent with the Shannon entropy of the coarse-grained binary projection of the stochastic ledger state. As the system evolves, entropy decreases across all regimes, reflecting the contractive effect of repeated applications of the transition kernel ($T_{\alpha }$). This decrease should be interpreted as an empirical statistical trend arising from the underlying stochastic process rather than a deterministic update rule.

### 6.2. Entropy Contraction Rate and Proposition 2

Proposition 2 characterizes the existence of a positive contraction rate (γ) governing the asymptotic decay of entropy under the induced Markov dynamics. The experimental results provide empirical evidence consistent with this prediction. For instance, under α=0.1, entropy decreases from approximately 0.469 to $5.85\times 10^{-5}$ over 99 blocks, indicating strong contraction behavior in the low-adversary regime.

As α increases, the decay becomes progressively slower, with α=0.3 reaching approximately $1.61\times 10^{-4}$ at block 99. This trend is consistent with the spectral interpretation of γ, suggesting that increased adversarial influence reduces the effective convergence speed of the induced Markov process. Rather than assuming strict exponential decay, the observed behavior is better understood as an asymptotic contraction process governed by the spectral properties of $T_{\alpha }$.

### 6.3. Tampering Effects and Theorem 3

The tampering experiments shown in Figure 3 and Figure 4 provide empirical validation of Theorem 3, which formalizes immutability in terms of persistent entropy deviations under adversarial perturbation.

At the tampering point, a discrete upward shift in entropy is observed across all values of α, reflecting an exogenous injection of uncertainty into the system. Importantly, this perturbation does not alter the transition kernel ($T_{\alpha }$) but modifies the realized state trajectory. After the perturbation, entropy continues to evolve under the same contractive dynamics; however, a long-lasting separation from the unperturbed trajectory is observed empirically.

A key observation is that the entropy gap between honest and tampered trajectories persists over a long horizon while gradually diminishing under the same stochastic operator. Although both trajectories continue to contract, the injected perturbation is only attenuated asymptotically rather than being eliminated immediately.

### 6.4. Off-Chain Entropy and Theorem 4

In contrast, the off-chain setting shown in Figure 2 validates Theorem 4. Here, data integrity is modeled as a Bernoulli random variable with fixed tampering probability, resulting in a static entropy measure without temporal evolution.

Unlike the on-chain case, the off-chain model lacks an endogenous contractive mechanism analogous to the Markov operator ($T_{\alpha }$). As a result, entropy does not evolve as a function of block height or time but, instead, remains a fixed functional of the underlying probability distribution. This highlights a fundamental structural difference: off-chain systems do not exhibit intrinsic entropy contraction, and any notion of stability must arise from external verification rather than internal stochastic dynamics.

### 6.5. Conceptual Link to Computational Entropy and BVM Expressiveness

The observed entropy dynamics can be interpreted in a broader computational context, as suggested by Theorems 1 and 2. While not establishing strict equivalence, the contraction behavior of on-chain entropy is conceptually analogous to repeated application of a contractive operator on a probabilistic state space, similar in nature to the Markov kernel ($T_{\alpha }$). In particular, the accumulation of consensus-driven updates in the blockchain resembles iterative stochastic transformations that progressively reduce uncertainty. This is consistent with the expressive capabilities of computational models such as a BVM, where state evolution encodes structured information transformations. From this perspective, entropy serves as an observable quantity reflecting the informational stability of an evolving stochastic computation process.

### 6.6. Synthesis and Implications

The results demonstrate that blockchain entropy provides a consistent and interpretable measure of uncertainty over a stochastic consensus system. The theoretical propositions are supported by empirical evidence showing contraction behavior in expectation, rate modulation consistent with adversarial influence, and persistent deviations under adversarial perturbation.

Importantly, in this framework, immutability should be understood as an emergent property of repeated stochastic contraction rather than a static cryptographic guarantee. The α parameter governs both the speed of convergence and the sensitivity to perturbations, thereby linking system-level adversarial strength with observable entropy dynamics.

The comparison with off-chain systems further highlights that entropy contraction is not a generic property of data storage but a consequence of consensus-induced stochastic dynamics. These findings unify spectral properties of Markov operators, stochastic process behavior, and entropy-based information flow into a coherent analytical framework for blockchain robustness.

### 6.7. Assumptions and Limitations

The objective of this work is to establish a general stochastic information-theoretic framework for analyzing blockchain consistency, entropy evolution, and adversarial uncertainty under abstract consensus assumptions. The present work does not attempt to derive the transition kernel ($T_{\alpha }$) from a specific blockchain implementation such as Bitcoin, Ethereum, or other concrete consensus protocols. Instead, the analysis is intentionally formulated at the level of an abstract stochastic consensus process satisfying asymptotic consistency, honest-majority concentration, and ergodic convergence properties.

Accordingly, the entropy quantities introduced in this paper should be interpreted as functionals over probability measures induced by ledger-state stochastic processes rather than deterministic properties of individual blockchain objects. The probability distributions are defined on the probability space of $\left(\Omega ,F,P_{\alpha }\right)$, where the stochastic process governing ledger evolution is induced by the consensus transition kernel ($T_{\alpha }$). This formulation allows entropy evolution to be analyzed within a mathematically consistent Markov-process framework.

The entropy decay model introduced in this work is therefore not intended as a protocol-specific law derived directly from implementation-level blockchain mechanics. Rather, the exponential decay behavior emerges under standard spectral-gap and geometric-ergodicity assumptions commonly used in stochastic process analysis. In particular, the parameter expressed as

$$\gamma =-\log\lambda_{2}\left(T_{\alpha }\right)$$

is interpreted as an abstract contraction characteristic of the induced Markov operator, where $\lambda_{2}\left(T_{\alpha }\right)$ denotes the second-largest eigenvalue magnitude. Consequently, γ should not be interpreted as a phenomenological fitting parameter imposed externally on the entropy trajectory. Instead, it represents a theoretical convergence descriptor associated with the stochastic dynamics of the consensus process.

At the same time, the present framework remains assumption-driven in several aspects. The analysis assumes the existence of an effective contraction mechanism under the honest-majority condition of α<τ, together with eventual probability concentration toward the dominant honest chain. These assumptions are standard in abstract consensus-security formulations, but the current work does not provide a protocol-level derivation of the spectral gap or ergodicity conditions from the detailed operational rules of specific blockchain systems. Therefore, the results should be interpreted as conditional theoretical statements within an abstract stochastic consensus framework rather than fully protocol-derived security theorems.

Similarly, the experimental evaluation should not be interpreted as a direct physical simulation of a production blockchain network. The experimental trajectories correspond to finite-sample Monte Carlo realizations of the induced stochastic process, together with empirical estimation of the transition operator (${\hat{T}}_{\alpha }$). The observed entropy contraction is therefore interpreted as an empirical manifestation of operator contraction behavior rather than as the fitting of the curve to a predefined exponential model. Nevertheless, the coarse-grained, two-state approximation adopted for visualization and entropy computation remains a simplified projection of the full ledger state space. The experiments are intended primarily to illustrate consistency between the stochastic information-theoretic framework and empirical entropy behavior rather than to reproduce all operational characteristics of real-world blockchain deployments.

Furthermore, while the current framework establishes a mathematically structured connection between entropy evolution and stochastic consensus dynamics, several aspects remain open for future investigation. In particular, future work may derive explicit transition kernels ($T_{\alpha }$) for concrete blockchain protocols, analyze protocol-dependent spectral gaps, study mixing-time behavior under realistic network latency and fork dynamics, and establish tighter bounds relating adversarial rewrite probabilities to entropy dissipation rates. Therefore, the present work should be understood as an abstract theoretical foundation connecting blockchain consensus, stochastic processes, and information-theoretic entropy rather than as a complete implementation-level security proof for any particular blockchain protocol.

## 7. Conclusions

In this work, we systematically investigated the role of entropy-based metrics in evaluating blockchain systems, focusing on both on-chain and off-chain data, as well as their application to a Blockchain Virtual Machine (BVM). Through a combination of theoretical analysis, numerical simulations, and probabilistic modeling, several key findings emerged. On-chain systems exhibit a progressive reduction in uncertainty, highlighting their robustness and reliability, whereas off-chain data cannot achieve the same level of uncertainty reduction. The BVM demonstrates predictable computational behaviors, providing a solid foundation for assessing smart-contract expressiveness and execution integrity.

The contributions in this paper are threefold. First, we establish a unified entropy-based framework to characterize immutability, integrity, and computational properties across blockchain environments. Second, we provide analytical and simulation-based evidence linking entropy dynamics to system security, resilience, and protocol efficiency. Third, we introduce practical metrics to monitor ledger health, detect tampering, and guide protocol design, thereby bridging theoretical foundations with operational applications.

By connecting probabilistic modeling, cryptographic security assumptions, and computational complexity, our framework offers a comprehensive lens for understanding blockchain behavior under varying adversarial conditions. This work not only deepens theoretical insights into blockchain dynamics but also provides actionable tools for protocol evaluation, smart-contract analysis, and system monitoring, supporting the development of secure and reliable distributed ledgers.

## Data Availability

The data supporting the findings of this study are available from the corresponding author upon reasonable request, subject to confidentiality and ethical restrictions.
